# Wm. R. Warner

**Published:** 1882-03-20

**Authors:** 


Wm. R. Warner's Pocket Drug Case.—The beautiful red Morocco Drug Case presented by the Professor of Physiology as a premium for the best examination in that branch at the Southern Medical College Commencement, was from the House of Wm. R. Warner & Co., Manufacturing Druggists, Philadelphia. It was filled with *parvules*, kindly sent for that purpose, by that excellent House. These parvules have proven a great convenience to practitioners who have tried them, being reliable as to purity, neat and beautiful in appearance, and in such minute fractional portions that the dose may be graded to any desired quantity without the trouble of compounding. Armed with these beautiful sugar-coated parvules the practitioner need not fear offending the most fastidious stomach, and can compete with the homeopath in respect to neatness and attractiveness of his remedies. We have for some time been using these parvules, and have found them not only a great convenience, but safe, reliable and efficient in action.

